# The Relationship Between Socioeconomic Status and CV Risk Factors: The CRONICAS Cohort Study of Peruvian Adults

**DOI:** 10.1016/j.gheart.2015.12.005

**Published:** 2016-03

**Authors:** Renato Quispe, Catherine P. Benziger, Juan Carlos Bazo-Alvarez, Laura D. Howe, William Checkley, Robert H. Gilman, Liam Smeeth, Antonio Bernabé-Ortiz, J. Jaime Miranda, CRONICAS Cohort Study Group, Antonio Bernabé-Ortiz, Juan P. Casas, George Davey Smith, Shah Ebrahim, Héctor H. García, Robert H. Gilman, Luis Huicho, Germán Málaga, J. Jaime Miranda, Víctor M. Montori, Liam Smeeth, William Checkley, Gregory B. Diette, Robert H. Gilman, Luis Huicho, Fabiola León-Velarde, María Rivera, Robert A. Wise, William Checkley, Héctor H. García, Robert H. Gilman, J. Jaime Miranda, Katherine Sacksteder

**Affiliations:** ∗ CRONICAS Center of Excellence in Chronic Diseases, Universidad Peruana Cayetano Heredia, Lima, Peru; † Division of Cardiology, University of Washington, Seattle, WA, USA; ‡ MRC Integrative Epidemiology Unit at the University of Bristol, School of Social and Community Medicine, University of Bristol, Bristol, United Kingdom; § Program in Global Disease Epidemiology and Control, Department of International Health, Bloomberg School of Public Health, Johns Hopkins University, Baltimore, MD, USA; ‖ Division of Pulmonary and Critical Care, School of Medicine Johns Hopkins University, Baltimore, MD, USA; ¶ Asociación Benéfica PRISMA, Lima, Peru; # Faculty of Epidemiology and Population Health, London School of Hygiene and Tropical Medicine, London, United Kingdom

## Abstract

**Background:**

Variations in the distribution of cardiovascular disease and risk factors by socioeconomic status (SES) have been described in affluent societies, yet a better understanding of these patterns is needed for most low- and middle-income countries.

**Objective:**

This study sought to describe the relationship between cardiovascular risk factors and SES using monthly family income, educational attainment, and assets index, in 4 Peruvian sites.

**Methods:**

Baseline data from an age- and sex-stratified random sample of participants, ages ≥35 years, from 4 Peruvian sites (CRONICAS Cohort Study, 2010) were used. The SES indicators considered were monthly family income (n = 3,220), educational attainment (n = 3,598), and assets index (n = 3,601). Behavioral risk factors included current tobacco use, alcohol drinking, physical activity, daily intake of fruits and vegetables, and no control of salt intake. Cardiometabolic risk factors included obesity, elevated waist circumference, hypertension, insulin resistance, diabetes mellitus, low high-density lipoprotein cholesterol, and high triglyceride levels.

**Results:**

In the overall population, 41.6% reported a monthly family income <US$198, and 45.6% had none or primary education. Important differences were noted between the socioeconomic indicators: for example, higher income and higher scores on an asset index were associated with greater risk of obesity, whereas higher levels of education were associated with lower risk of obesity. In contrast, higher SES according to all 3 indicators was associated with higher levels of triglycerides.

**Conclusions:**

The association between SES and cardiometabolic risk factors varies depending on the SES indicator used. These results highlight the need to contextualize risk factors by socioeconomic groups in Latin American settings.

## Highlights

• The relationship between socioeconomic status (SES) and risk factors for cardiovascular disease depends on the indicator used.
• High SES groups, based on income level, had a higher prevalence of elevated waist circumference (WC) and obesity than did the low-income group.
• High SES groups, based on educational attainment, had higher prevalence of elevated triglycerides but lower prevalence of obesity than did low-education groups.
• High SES groups, based on assets index, had higher prevalence of elevated WC, elevated triglycerides, and insulin resistance.

The negative effects of urbanization and unhealthy lifestyles along with population aging are particularly challenging for low- and middle-income countries (LMIC) 1, 2, 3, 4. Variations in the distribution of cardiovascular disease and risk factors by socioeconomic status (SES) have been described in high-income countries. However, a better understanding of these patterns is needed for most LMIC as the prevalence of risk factors for cardiovascular disease, such as obesity, hypertension, or diabetes mellitus, is increasing in Latin America 5, 6, 7, 8.

Previous studies have found that the prevalence of dietary habits 9, 10, cardiometabolic risk factors, and cardiovascular events 11, 12, 13, 14, 15, 16, 17, 18, 19, 20, 21, 22, 23, 24, 25, 26, 27, 28, 29 vary across sociodemographic groups and by the country's Human Development Index. For example, in high-income countries, there is a negative association between obesity and higher income and educational attainment, whereas in low-income countries, there is a positive association between education and obesity 17, 21.

In Peru, over one-third of the population lives in the large urban capital, Lima, but a substantial population still lives in rural areas where access to resources, including health care and education, are limited. These rural populations are often poorer and less educated than the urban areas with limited access to medical care and chronic disease treatment [30]. The impact of these sociodemographic factors, together with urbanization and geographical features, for example, populations residing at high altitudes, and the prevalence of cardiovascular disease are not well established 1, 31, 32, 33. Previous studies in Peru have reported associations between different SES indicators and cardiometabolic risk factors 32, 34, 35, 36; however, the majority of them focused on single settings. For example, 1 study found wealthier women were more likely to be obese, and this association was stronger in rural areas [34]. Conversely, more educated women were less likely to be obese, especially in urban areas 34, 36. Nonetheless, a better understanding of these patterns is needed for most LMIC.

This study aimed to determine the association among 3 indicators of SES (monthly family income, educational attainment, and assets index) with behavioral and cardiometabolic risk factors in a Peruvian population.

## Methods

### Study design, setting, and participants

We used baseline data from the CRONICAS Cohort Study, conducted by CRONICAS Center of Excellence in Chronic Diseases [37], which was originally designed to investigate the prevalence of cardiovascular and chronic pulmonary diseases and its progression in 4 different rural/urban and coastal/high-altitude Peruvian settings [38]. Individuals ages ≥35 years who were full-time residents in the area, able to understand procedures, and provide informed consent were invited to participate in the study. We identified a sex-and-age stratified random sample (35 to 44, 45 to 54, 55 to 64, and ≥65 years) of eligible subjects and enrolled only 1 participant per household. In Puno, recruitment was stratified by location (urban or rural). Recruitment began in September 2010 and was finished once 1,000 participants per site were enrolled [38]. Baseline data from 2010 was used for this study and analyzed in 2015.

### Data collection

A team of community health workers was trained to enroll participants and to conduct household questionnaires assessing sociodemographic and behavioral variables. Participants were invited to a clinic visit where standing and sitting height, weight, and waist circumference where measured in triplicate using standardized techniques. Systolic and diastolic blood pressure were also measured in triplicate using an automatic monitor (OMRON HEM-780, Omron Healthcare, Hoffman Estates, IL, USA) previously validated for an adult population [39]. In this study, we used the mean of the second and third measurements. Fasting blood samples were obtained using standardized methods and calibrated tools [38]. Total cholesterol, triglycerides, high-density lipoprotein cholesterol (HDL-C), and insulin were measured in serum, whereas fasting glucose was assessed in plasma using an enzymatic colorimetric method (GOD-PAP, Modular P-E/Roche-Cobas, Germany). Triglycerides and HDL-C were measured using a Cobas Modular Platform automated analyzer and reagents supplied by Roche Diagnostics (Basel, Switzerland). All samples were analyzed in a single facility, and, for quality assurance, the quality of assays was checked with regular external standards and internal duplicate assays and monitored by BioRad (Hercules, CA, USA).

### Study variables

We evaluated sociodemographic, behavioral, and cardiometabolic variables (definitions are shown in Table 1 40, 41). SES was approached through 3 indicators: 1) educational attainment: none or primary education, secondary, and higher; 2) assets index [40]: estimated based on the number of possessions for each individual, tertiles were calculated for each site, separately; and 3) monthly family income: up to PEN 550 (<US$198), PEN 551 to 1,500 (US$199 to 540), and PEN >1,500 (≥US$541); in 2010, the minimal wage in Peru was PEN 550, and the exchange rate was US$1 = PEN 2.78. Demographic information included age, sex, and study site. Behavioral risk factors included current tobacco use, hazardous alcohol drinking (based on the validated Alcohol Use Disorders Identification Test [AUDIT] score), leisure-time physical activity, daily intake of fruits and vegetables, and salt intake. Cardiometabolic risk factors included obesity (body mass index >30 kg/m^2^), elevated waist circumference (WC), hypertension, insulin resistance, diabetes mellitus, low HDL-C, and elevated triglycerides (TG).

**Table 1:** Definition of sociodemographic, behavioral, and cardiometabolic variables

Sociodemographic and behavioral factors
Age groups: 35–44, 45–54, 55–64, ≥65 yrs
Education: none or primary, secondary, and higher
Assets index: divided into tertiles, calculated for each site based on number of possessions [40]
Site: Lima (costal urban), Puno urban (high-altitude urban), Puno rural (high-altitude rural), and Tumbes (costal rural)
Current tobacco use: self-reported (“Which best describes your history of smoking: never user or former user/current user?”)
Alcohol drinking: the Alcohol Use Disorders Identification Test (AUDIT) score ≥8 points for hazardous drinking, which is a pattern of alcohol consumption that increases the risk of harmful consequences for the user or others [41]
Leisure-time physical activity: days of moderate and vigorous physical activity in leisure-time during the last 7 days
Salt intake: lack of control of salt or sodium intake (“Do you do something regularly to control your salt or sodium intake? Yes/No”)
Cardiometabolic abnormalities considered
Hypertension: SBP ≥140 mm Hg or DBP ≥90 mm Hg, or antihypertensive medication, or physician diagnosis
Hypertriglyceridemia: fasting triglyceride ≥150 mg/dl
Low HDL-C level: HDL-C <40 mg/dl in men <50 mg/dl in women
Diabetes mellitus: fasting glucose ≥126 mg/dl, or glucose-lowering medication, or self-reported diagnosis
Insulin resistance: HOMA-IR >5.00 (>90th percentile)
Obesity: BMI ≥30 kg/m^2^
Waist circumference: ≥90 cm in men or ≥80 cm in women

BMI, body mass index; DBP, diastolic blood pressure; HDL-C, high-density lipoprotein cholesterol; HOMA-IR, homeostasis model assessment of insulin resistance; SBP, systolic blood pressure.

### Statistical analysis

Each SES indicator was divided into 3 categories. Sociodemographic, behavioral, and cardiometabolic variables were described according to each SES indicator. Means ± SD and proportions were compared by analysis of variance and chi-square tests, respectively. To determine association between behavioral/cardiometabolic risk factors and SES indicators, adjusted by age, sex, study site, and SES indicators, where appropriate, prevalence ratios were estimated using log-Poisson models with robust estimations. For all analyses, we estimated 95% confidence intervals and considered p < 0.05 as statistically significant. We used Stata (version 12.0, College Station, TX, USA) for all analyses.

### Ethics

All participants provided verbal informed consent after our research team read the entire informed consent document to them and any questions were answered. Informed consents were verbally obtained because of high illiteracy rates. The study was approved by the institutional review boards of Universidad Peruana Cayetano Heredia and A.B. PRISMA, in Lima, Peru, and at the Bloomberg School of Public Health, Johns Hopkins University, in Baltimore, MD, USA.

## Results

A total of 3,619 individuals were enrolled in the baseline survey of CRONICAS Cohort Study, and 3,220, 3,601, and 3,598 participants had complete information about monthly family income, assets index, and educational attainment, respectively, besides complete information about behavioral and cardiometabolic risk factors. Cronbach alpha of 0.64 and average interitem correlation of 0.37 showed low internal consistency among SES indicators. In general, the largest proportions of population belonged to the lowest categories of SES indicators: 41.6% reported a monthly family income <US$198, and 45.6% had none or primary education. Additionally, we observed that most individuals within the lowest assets index tertile and lowest monthly family income were also in the group of lowest educational attainment.

### Cardiovascular risk factors by SES indicators

Individuals in the lowest income group were older than those in the highest income group. Compared with individuals with family income of <US$198 and US$199 to US$540, those with family income of ≥US$541 presented, overall, a less favorable cardiovascular risk profile characterized by higher proportions of behavioral (current tobacco use and alcohol intake) and cardiometabolic risk factors, such as obesity and high TG (p < 0.001) (Table 2).

**Table 2:** Baseline sociodemographic, behavioral, and clinical information by monthly family income (n = 3,220)

	<US$198 (n = 1,338)	US$199–540 (n=1,576)	≥US$541 (n = 306)	p Value
Sociodemographic characteristics				
Male	543 (40.7)	841 (53.4)	209 (68.3)	<0.001
Age, yrs	58.2 ± 13.5	53.7 ± 11.7	51.4 ± 10.6	<0.001
Age groups, yrs				
35–44	281 (20.9)	420 (26.5)	100 (32.4)	<0.001
45–54	280 (21.1)	456 (29.1)	100 (32.7)	
55–64	327 (24.5)	412 (26.1)	71 (23.5)	
≥65	447 (33.5)	288 (18.3)	35 (11.4)	
Educational attainment				
None or primary	869 (65.0)	506 (32.1)	47 (15.4)	<0.001
Secondary	367 (27.5)	609 (38.6)	75 (24.3)	
Higher	101 (7.5)	460 (29.3)	183 (60.3)	
Assets index tertiles				
Lowest	666 (49.8)	398 (25.2)	21 (6.9)	<0.001
Middle	421 (31.4)	578 (36.7)	64 (20.9)	
Highest	251 (18.8)	600 (38.1)	221 (72.2)	
Study site				
Lima	213 (15.9)	700 (44.4)	139 (45.4)	<0.001
Puno (urban)	188 (14.1)	337 (21.4)	113 (36.9)	
Puno (rural)	442 (37.0)	64 (30.1)	3 (16.7)	
Tumbes	495 (33.0)	475 (4.1)	51 (1.0)	
Behavioral risk factors				
Current tobacco use	95 (7.1)	228 (14.5)	55 (18.0)	<0.001
Alcohol drinking	153 (11.4)	248 (15.7)	66 (21.6)	<0.001
Physical activity in leisure time, days				
Moderate	0.2 ± 0.97	0.3 ± 1.12	0.4 ± 1.24	<0.001
Vigorous	0.1 ± 0.7	0.2 ± 0.97	0.4 ± 1.19	<0.001
Daily intake				
Fruit	0.6 ± 0.63	0.8 ± 0.7	1.1 ± 0.91	<0.001
Vegetables	0.3 ± 0.48	0.4 ± 0.41	0.5 ± 0.46	<0.001
No control of salt intake	1,196 (89.4)	1,369 (87.0)	261 (85.3)	<0.001
Cardiometabolic risk factors				
Obesity	290 (24.4)	442 (30.5)	88 (30.9)	<0.001
WC	90.4 ± 11.8	93.2 ± 10.1	94.7 ± 10.4	<0.001
Hypertension	375 (31.1)	385 (26.2)	77 (26.9)	0.01
Insulin resistance	98 (8.5)	168 (11.8)	31 (11.2)	0.02
Diabetes	108 (9.3)	122 (8.6)	23 (8.3)	0.737
Low HDL-C	732 (63.5)	953 (67.1)	193 (69.7)	0.059
High TG	416 (36.0)	677 (47.7)	140 (50.5)	<0.001

Values are n (%) or mean ± SD. The p values are for comparison within each SES indicator.

HDL-C, high density lipoprotein-cholesterol; SES, socioeconomic status; TG, triglycerides; WC, waist circumference.

People in the highest education group were younger than those in the lowest education group. Individuals with highest educational attainment (higher) presented higher physical activity, intake of fruits and vegetables, and control of salt intake, but also higher rates of current tobacco use and alcohol drinking, than those in lower education groups. Additionally, prevalence of cardiometabolic risk factors (hypertension, diabetes, and insulin resistance) was lower in individuals from highest compared with those from lowest education groups (p < 0.001) (Table 3).

**Table 3:** Baseline sociodemographic, behavioral, and clinical information by educational attainment (n = 3,598)

	None or primary (n = 1,642)	Secondary (n = 1,154)	Higher (n = 802)	p Value
Sociodemographic characteristics				
Male	606 (37.0)	656 (56.9)	481 (27.7)	<0.001
Age, yrs	61.7 ± 12.4	51.1 ± 10.8	50.5 ± 10.3	<0.001
Age groups, yrs				
35–44	178 (10.8)	392 (34.0)	281 (35.0)	<0.001
45–54	320 (19.5)	345 (29.9)	262 (32.7)	
55–64	457 (27.8)	276 (23.9)	184 (22.9)	
≥65	684 (41.9)	141 (12.2)	75 (9.4)	
Family income (dollars)				
<US$198	869 (61.2)	368 (35.1)	100 (13.4)	<0.001
US$199–540	505 (35.5)	608 (57.9)	462 (61.9)	
≥US$541	47 (3.3)	74 (7.0)	184 (24.7)	
Assets index tertiles				
Lowest	784 (47.8)	356 (30.8)	114 (14.2)	<0.001
Middle	542 (33.0)	384 (33.3)	252 (31.4)	
Highest	316 (19.2)	414 (35.9)	436 (54.4)	
Study site				
Lima	474 (28.8)	440 (38.1)	191 (23.8)	<0.001
Puno (urban)	140 (8.5)	203 (17.6)	420 (52.4)	
Puno (rural)	455 (27.7)	204 (17.7)	41 (5.1)	
Tumbes	574 (35.0)	307 (26.6)	150 (18.7)	
Behavioral risk factors				
Current tobacco use	114 (7.0)	161 (14.0)	123 (15.4)	<0.001
Alcohol drinking	145 (8.8)	213 (18.5)	159 (19.8)	<0.001
Physical activity in leisure time, days				
Moderate	0.16 ± 0.9	0.24 ± 1.0	0.45 ± 1.3	<0.001
Vigorous	0.09 ± 0.6	0.19 ± 0.8	0.46 ± 1.4	<0.001
Daily intake				
Fruit	0.62 ± 0.6	0.82 ± 0.8	0.88 ± 0.7	<0.001
Vegetables	0.33 ± 0.5	0.41 ± 0.4	0.45 ± 0.4	0.05
No control of salt intake	1,479 (90.0)	1,040 (89.9)	665 (83.6)	<0.001
Cardiometabolic risk factors				
Obesity	413 (27.8)	282 (27.0)	173 (25.1)	0.423
WC	90.81 ± 12.0	92.29 ± 10.6	93.06 ± 9.5	<0.001
Hypertension	499 (33.3)	237 (22.3)	165 (23.5)	<0.001
Insulin resistance	159 (11.1)	98 (9.6)	54 (8.1)	0.006
Diabetes	147 (10.3)	79 (7.6)	46 (6.9)	0.013
Low HDL-C	933 (65.3)	666 (65.0)	458 (69.0)	0.186
High TG	540 (37.8)	442 (43.1)	354 (53.3)	<0.001

Values are n (%) or mean ± SD. The p values are for comparison within each SES indicator.

Abbreviations as in Table 2.

Table 4 shows the baseline sociodemographic and clinical variables by assets index. Individuals were older in the lowest asset group compared with highest groups. Individuals within the highest assets index had higher proportions of current tobacco use and hazardous alcohol drinking, but higher physical activity and daily intake of fruit and vegetables than individuals in lower tertiles (p < 0.001). Cardiometabolic risk factors did not show significant differences, except from higher WC and greater proportion of high TG in individuals within the highest assets tertile compared with those within lower assets tertiles (p < 0.001).

**Table 4:** Baseline sociodemographic, behavioral, and clinical information by tertiles of assets index (n = 3,601)

	Lowest (n = 1,255)	Middle (n = 1,178)	Highest (n = 1,168)	p Value
Sociodemographic characteristics				
Male	476 (38.0)	592 (50.3)	677 (58.0)	<0.001
Age, yrs	58.47 (13.5)	55.53 (12.46)	53.16 (11.27)	<0.001
Age groups, yrs				
35–44	266 (21.0)	285 (24.1)	307 (26.1)	<0.001
45–54	241 (19.4)	309 (26.4)	372 (31.9)	
55–64	303 (24.2)	305 (25.8)	309 (26.5)	
≥65	442 (35.4)	278 (23.7)	180 (15.5)	
Family income				
<US$198	666 (61.4)	421 (39.6)	251 (23.4)	<0.001
US$199–540	398 (36.7)	578 (54.4)	600 (56.0)	
≥US$541	21 (1.9)	64 (6.0)	221 (20.6)	
Educational attainment				
None or primary education	784 (62.5)	543 (46.0)	316 (27.1)	<0.001
Secondary	355 (28.4)	382 (32.6)	418 (35.5)	
Higher	115 (9.1)	253 (21.4)	432 (37.4)	
Study site				
Lima	377 (30.0)	360 (30.6)	368 (31.5)	<0.001
Puno (urban)	256 (20.4)	254 (21.5)	254 (21.8)	
Puno (rural)	266 (21.2)	214 (18.2)	220 (27.9)	
Tumbes	356 (28.4)	350 (29.7)	326 (18.8)	
Behavioral risk factors				
Current tobacco use	97 (7.7)	138 (11.7)	164 (14.0)	<0.001
Alcohol drinking	122 (9.7)	183 (15.5)	213 (18.2)	<0.001
Physical activity in leisure time, days				
Moderate	0.17 ± 0.9	0.26 ± 1.1	0.33 ± 1.2	<0.001
Vigorous	0.08 ± 0.5	0.23 ± 1.0	0.31 ± 1.1	<0.001
Daily intake				
Fruit	0.63 ± 0.6	0.73 ± 0.7	0.87 ± 0.8	<0.001
Vegetables	0.33 ± 0.4	0.38 ± 0.4	0.45 ± 0.5	<0.001
No control of salt intake	1,125 (89.6)	1,037 (88.1)	1,025 (87.8)	0.317
Cardiometabolic risk factors				
Obesity	277 (25.2)	300 (28.5)	291 (27.2)	0.219
WC	89.69 ± 11.89	92.59 ± 10.92	93.11 ± 10.11	<0.001
Hypertension	329 (29.6)	279 (26.1)	293 (27.0)	0.175
Insulin resistance	89 (8.5)	112 (10.9)	110 (10.5)	0.136
Diabetes	90 (8.6)	92 (8.9)	90 (8.6)	0.937
Low HDL-C	682 (65.1)	677 (66.2)	699 (66.7)	0.726
High TG	402 (38.3)	430 (42.0)	504 (48.1)	<0.001

The assets index is based on number of possessions (13 in total). Values are n (%) or mean ± SD. The p values are for comparison within each SES indicator.

Abbreviations as in Table 2.

### Associations between socioeconomic status indicators and cardiovascular risk factors

Compared with the lowest category of family income, individuals within the highest group were found to be positively associated with obesity and elevated WC, whereas the middle SES group had a significant positive association with elevated TG and insulin resistance. Higher educational attainment was negatively associated with obesity, but positively associated with high TG, comparing with individuals with none or primary education. Higher levels of assets were positively associated with elevated WC, high TG, and insulin resistance, whereas the middle tertile was positively associated with obesity, elevated WC, and insulin resistance compared with those within the lowest tertile of assets index. Individuals with higher educational attainment had lower risk of lack of control of salt intake, compared with those with none or primary education. None of the SES indicators evaluated were associated with diabetes mellitus, low HDL-C, current tobacco use, or alcohol drinking after adjustment for age, sex, study site, and other SES indicators (Table 5). Additional models were constructed adjusting separately for each SES indicator and did not alter our observations. In addition, colinearity was nonexistent (Online Table 1).

**Table 5:** Prevalence ratios between cardiovascular risk factors and SES indicators

	Monthly Family Income			Educational Attainment			Assets Index		
	<US$198	US$199–540	≥US$541	None or Primary	Secondary	Higher	Lowest	Middle	Highest
Obesity	Ref	1.11 (0.96–1.28)	**1.29 (1.02–1.62)**	Ref	0.89 (0.77–1.03)	**0.76 (0.62–0.92)**	Ref	**1.17 (1.02–1.35)**	1.14 (0.98–1.34)
Elevated WC	Ref	1.03 (0.99–1.09)	**1.12 (1.03–1.21)**	Ref	1.02 (0.97–1.08)	1.02 (0.96–1.09)	Ref	**1.08 (1.02–1.13)**	**1.12 (1.06–1.18)**
Hypertension	Ref	0.88 (0.77–1.01)	0.95 (0.75–1.20)	Ref	0.94 (0.80–1.10)	1.01 (0.82–1.22)	Ref	0.95 (0.83–1.10)	1.13 (0.97–1.30)
Low HDL-C	Ref	1.01 (0.95–1.18)	1.07 (0.97–1.19)	Ref	0.98 (0.92–1.05)	1.01 (0.93–1.10)	Ref	1.02 (0.95–1.09)	1.05 (0.97–1.12)
High TG	Ref	**1.13 (1.01–1.26)**	1.10 (0.93–1.30)	Ref	1.09 (0.97–1.21)	**1.22 (1.07–1.39)**	Ref	1.03 (0.92–1.15)	**1.15 (1.02–1.29)**
Diabetes mellitus	Ref	0.94 (0.70–1.25)	1.01 (0.62–1.66)	Ref	0.96 (0.70–1.31)	0.70 (0.44–1.10)	Ref	1.17 (0.87–1.57)	1.33 (0.97–1.83)
Insulin resistance	Ref	**1.30 (1.01–1.67)**	1.51 (0.98–2.32)	Ref	0.86 (0.66–1.13)	0.75 (0.53–1.05)	Ref	**1.34 (1.02–1.75)**	**1.40 (1.06–1.88)**
Current smoker	Ref	1.24 (0.96–1.58)	1.18 (0.82–1.70)	Ref	0.98 (0.77–1.27)	0.98 (0.73–1.30)	Ref	1.17 (0.92–1.50)	1.21 (0.95–1.56)
Alcohol drinking	Ref	1.06 (0.83–1.35)	1.10 (0.77–1.60)	Ref	1.12 (0.88–1.44)	0.88 (0.65–1.19)	Ref	1.16 (0.89–1.50)	1.03 (0.79–1.36)
No control of salt intake	Ref	0.99 (0.96–1.02)	0.98 (0.92–1.04)	Ref	0.98 (0.94–1.01)	**0.93 (0.88–0.97)**	Ref	0.99 (0.96–1.02)	0.99 (0.96–1.03)

Values are PR (95% CI). Bold = p < 0.05. Regression models (binary outcomes) were adjusted by age, sex, study site, assets index, family income, and education attainment, where appropriate. Alcohol drinking was defined as AUDIT score ≥8 points for hazardous drinking.

CI, confidence interval; PR, prevalence ratio; Ref, reference; other abbreviations as in Table 2.

## Discussion

Our study found that the association between SES and cardiometabolic risk factors varies depending on the SES indicator used, even after adjusting for age, sex, study site, and SES indicators. These findings highlight the need to contextualize risk factors by socioeconomic groups in Latin American settings and strongly suggest that SES factors driving cardiovascular risk operate through different pathways. We found that the prevalence of obesity was higher in those with higher family income level and in the middle assets tertile, whereas educational attainment seemingly had a protective effect, suggesting that pathways toward obesity are complex.

In our study, we used 3 SES indicators. Education attempts to capture the knowledge-related assets of a person; it is thought to measure early life SES given that education is usually completed in young adulthood and strongly determined by parental characteristics. Assets and family income represent and measure the material resources component and can influence a wide range of material circumstances with direct implications for health [42]. SES is well-documented and easy to measure in high-income countries but less is known and understood in LMIC settings and, as a result, it is measured in many different ways depending on the epidemiological study and location [43]. The relationships among family income, assets, and education and SES, a product of material and social factors, vary by country and within regions depending largely on the country's Human Development Index 21, 44. No single measure of SES is ideal for all settings, especially in developing countries [43].

### Comparison with previous studies

We found a positive association among obesity and family income level and middle assets index, but negative with educational attainment; however, these results are not entirely consistent with previous studies. This may be explained in part because these SES indicators may have differing trends with obesity depending on the country and its Human Development Index [17]. For instance, in low-income countries, the more affluent, as well as those with higher educational attainment, were more likely to be obese 17, 21. However, in middle-income countries, among women with none or primary education, the effect of income with obesity was positive; whereas, among women with higher education, the effect was either absent or negative [17]. In LMIC, possessions and wealth index were positively associated with obesity in Peru 34, 45, and Colombia [46]. However, there were inconsistent results with regard to the association between education and obesity, being negative in Brazil [47] and Peru [37], but positive in Uganda [16] and South Asia [28]. Our results also show a negative association between high educational attainment and obesity, which is consistent with other Peruvian studies, where there was a negative association with obesity in women with higher education 34, 36 and may reflect what is seen in developed countries, where the burden of disease transitions from initially higher SES groups to lower as the country becomes more developed.

Insulin resistance and hypertriglyceridemia are common comorbidities of obesity [48]. In our study, we found a positive association between highest assets tertile and high educational attainment with high TG, as well as a positive association between the middle SES group of family income and both middle and highest assets tertiles with insulin resistance. This is consistent with previous studies that have found a positive association with higher SES and high TG 11, 14, 19, 22, 26, 28. Of note, we did not find any association with total cholesterol and low HDL-C, which could be due to the high baseline prevalence of low HDL-C in the overall population. It is interesting that we found an association with metabolic disorders, elevated TG, and insulin resistance in the middle-income group and not in the high-income group because we had more obesity in the high income group. However, assets index may be a better variable for SES and is more consistent with both middle and highest assets tertile having strong associations with insulin resistance and elevated WC.

We found no association between any SES indicator and hypertension or diabetes mellitus after adjustment for age, sex, and the other SES indicators. This may reflect the fact that the highest SES groups were generally younger than the lowest SES groups (by up to 10 years). Conversely, many previous studies in LMIC have demonstrated that hypertension and diabetes are positively associated with income 12, 14, 18, 20, 24. Although we did not find an association, there may be alternative explanations. For example, South Korea and China, which have become more developed recently, found that hypertension [27] and diabetes [24] had a negative association with income and education level. Similar to obesity, this trend is seen in developed countries, where the burden of these diseases transitions from the higher SES groups to lower as the country becomes more developed 1, 25, 49. Our study may be evidence of that transition occurring in Peru.

Current tobacco use and higher alcohol consumption are generally associated with lower education levels, but this association is not consistent among other SES indicators 14, 16, 19, 22, 28. In our study, we did not find a significant association between current tobacco use or alcohol consumption and SES indicators; however, we did observe higher proportions of current tobacco use and alcohol consumption in individuals within the highest educational attainment and assets index, compared with counterparts from lower SES categories. These groups were also significantly younger as well. Previous studies have also found an association between SES and healthier diet with higher fruit and vegetable consumption among those in the higher SES groups 9, 11, 28, 44, as well as higher consumption of whole grains, lean meats, fish, and low-fat dairy, as compared to lower SES groups who consumed more refined grains, lower fruits and vegetables, and added fats 9, 10. Individuals with highest educational attainment had lower risk for having lack of control of salt intake compared with those with none or primary education, similar to what was found in developed countries such as Great Britain, Japan, and Australia 50, 51, 52.

### Complexity of socioeconomic drivers of obesity

We observed a negative association between obesity and education, but positive with assets index and family income. Increasing wealth in populations from LMIC have been shown to promote poor dietary habits [53] and may also result in a decrease in physical activity, consequently leading to higher risk of obesity [54]. On the other hand, knowledge and skills attained through education make individuals more receptive to health education messages [42]. During the last decades, there have been aggressive food marketing campaigns in LMIC, especially for fast food, and new consumers require greater cognitive skills to deal with these sophisticated economic signals. Given the high exposure to these energy-dense, processed foods that goes along with higher wealth, a rise in obesity prevalence is expected across all socioeconomic strata, but especially among lower educated individuals [17]. This exposure may occur before public health educating initiatives are appropriately and broadly implemented, creating a gap between obesogenic effect of wealth and protective effect of education, resulting in a higher incidence of obesity. In this context, the beneficial effect of education may result from assisting in correcting cognitive biases created through marketing, leading to a better perception of risk, as well as cognitive abilities that influence health decisions [53]. For instance, 2 studies of cash transfer programs in Mexico and Colombia showed that higher incomes increased fat and sugar consumption, especially when adult education sessions were not considered within these programs 55, 56.

Another potential explanation is that individuals with higher levels of education may come from more progressive families, thereby adopting norms and social lifestyles of developed countries, including a higher consideration for slim body shapes, which might derive in more physical activity and healthier diets.

### Public health implications

Understanding the sociodemographic patterning of risk factors is important from a public health perspective for better characterization of the social distribution of health and hence for planning of prevention programs [57]. Our findings suggest that it would not be straightforward to design obesity-prevention interventions targeted at at-risk socioeconomic groups in Peru, because we observed negative associations for education but positive associations for wealth. As the Peruvian economy continues to develop over time, it will be important to monitor whether these associations change. Based on other middle-income countries, we may expect the association for wealth to reverse, such that lower wealth groups (in addition to lower education groups) have a higher risk of obesity. In fact, the PERU MIGRANT (Peru's Rural to Urban MIGRANTs) study showed that individuals at the top tertile of assets index were at higher risk of developing central obesity, compared with the bottom tertile after 5 years of follow-up [58].

### Study limitations

This study has some limitations that must be considered. Our study groups come from 4 different regions, which may not be representative of the whole population in Peru. Despite this, our study offers unique insights into a combination of rural and urban sites located at low- and high-altitude settings. Our cross-sectional approach prevents interpretation of causal relationships between SES indicators and cardiovascular risk factors. Although a standardized protocol was used, some of the variables, such as history of diabetes or hypertension, were self-reported and prone to recall bias together with limitations of availability of health services and availability of diagnosis. However, a composite definition for those variables was used in this study, which included glucose and blood pressure measurements. Finally, participant's response rate was high when analyzed by SES indicators across sites except from rural Puno (Online Table 2). In this site we observed a lower response rate for monthly family income compared with other study sites. However, further sensitivity analyses comparing those with and without data on family income showed no clear differences (Online Tables 3 and 4).

## Conclusions

Our study found that the association between SES and cardiometabolic risk factors varies depending on the SES indicator used. A significantly positive association was observed only between obesity and elevated WC and family income and assets index, whereas educational attainment had a protective effect. We may hypothesize that vascular risk varies by different SES measures, suggesting that SES factors driving cardiovascular risk, especially through obesity, are operating through different pathways. These results highlight the need for careful monitoring and to contextualize risk factors by socioeconomic groups in developing countries in Latin America, with a focus on high-risk groups to decrease development of cardiovascular disease.

## Figures and Tables

**Online Table 1:** PR between cardiovascular risk factors and SES indicators

	Monthly Family Income∗			Educational Attainment†			Assets Index‡		
	<US$198	US$199–540	≥US$541	None or Primary	Secondary	Higher	Lowest	Middle	Highest
Obesity	Ref	1.10 (0.97–1.26)	**1.25 (1.01–1.54)**	Ref	0.93 (0.81–1.07)	**0.83 (0.69–0.98)**	Ref	1.14 (1.00–1.31)	1.13 (0.99–1.30)
Elevated WC	Ref	**1.07 (1.02–1.12)**	**1.18 (1.10–1.27)**	Ref	1.05 (0.99–1.10)	**1.09 (1.02–1.15)**	Ref	**1.10 (1.05–1.16)**	**1.15 (1.10–1.21)**
Hypertension	Ref	0.90 (0.80–1.03)	1.03 (0.83–1.28)	Ref	0.97 (0.84–1.12)	1.07 (0.89–1.28)	Ref	0.96 (0.84–1.10)	1.10 (0.96–1.25)
Low HDL-C	Ref	1.03 (0.96–1.09)	**1.10 (1.01–1.21)**	Ref	0.99 (0.93–1.05)	1.04 (0.96–1.12)	Ref	1.03 (0.97–1.09)	1.05 (0.99–1.12)
High TG	Ref	**1.22 (1.10–1.35)**	**1.27 (1.09–1.48)**	Ref	**1.13 (1.01–1.25)**	**1.37 (1.21–1.54)**	Ref	1.08 (0.97–1.20)	1.23 (1.11–1.36)
Diabetes mellitus	Ref	0.96 (0.73–1.27)	1.04 (0.66–1.65)	Ref	0.95 (0.70–1.29)	0.82 (0.55–1.22)	Ref	1.10 (0.83–1.46)	1.16 (0.88–1.54)
Insulin resistance	Ref	**1.34 (1.05–1.71)**	**1.56 (1.06–2.31)**	Ref	0.91 (0.70–1.19)	0.84 (0.61–1.18)	Ref	**1.32 (1.02–1.71)**	**1.37 (1.05–1.78)**
Current smoker	Ref	**1.29 (1.02–1.64)**	1.30 (0.93–1.81)	Ref	1.03 (0.81–1.32)	1.13 (0.86–1.50)	Ref	1.22 (0.96–1.54)	**1.28 (1.02–1.60)**
Alcohol drinking	Ref	1.05 (0.84–1.32)	1.01 (0.72–1.42)	Ref	1.15 (0.90–1.46)	0.93 (0.70–1.24)	Ref	**1.35 (1.05–1.74)**	1.23 (0.95–1.57)
No control of salt intake	Ref	0.97 (0.94–0.99)	0.95 (0.90–1.00)	Ref	0.97 (0.95–1.00)	**0.91 (0.87–0.95)**	Ref	0.97 (0.95–1.00)	**0.96 (0.94–0.99)**

Values are PR (95% CI). Bold = p < 0.05.

CI, confidence interval; HDL-C, high-density lipoprotein cholesterol; PR, prevalence ratio; Ref, reference; SES, socioeconomic status; TG, triglycerides; WC, waist circumference.

∗ Regression model was adjusted by age, sex, study site, and monthly family income.

† Regression model was adjusted by age, sex, study site, and educational attainment.

‡ Regression model was adjusted by age, sex, study site, and assets index.

**Online Table 2:** Response rate of individuals per SES indicator

	All Sites	Lima	Urban Puno	Rural Puno	Tumbes
Monthly family income	3,220 (89.2)	1,052 (94.6)	638 (83.5)	509 (72.7)	1,021 (98.9)
Educational attainment	3,598 (99.7)	1,104 (99.3)	763 (99.9)	700 (100)	1,031 (99.9)
Assets index	3,601 (99.8)	1,105 (99.4)	764 (100)	700 (100)	1,032 (100)
Total	3,608	1,112	764	700	1,032

Values are n (%) and n.

SES, socioeconomic status.

**Online Table 3:** Sensitivity analysis, rural Puno with data on family income (n = 509)

	Educational Attainment			Assets Index		
	None or Primary	Secondary	Higher	Lowest	Middle	Highest
Obesity	Ref	1.20 (0.61–2.36)	0.93 (0.22–3.81)	Ref	1.05 (0.56–1.95)	0.85 (0.40–1.82)
Elevated WC	Ref	1.27 (1.00–1.62)	1.41 (0.96–2.08)	Ref	1.21 (0.93–1.57)	**1.33 (1.01–1.75)**
Hypertension	Ref	1.03 (0.61–1.75)	0.88 (0.40–1.96)	Ref	0.84 (0.49–1.42)	1.03 (0.59–1.79)
Low HDL-C	Ref	1.12 (0.92–1.38)	**1.48 (1.02–2.15)**	Ref	1.06 (0.87–1.30)	1.04 (0.82–1.31)
High TG	Ref	0.91 (0.62–1.33)	1.23 (0.72–2.07)	Ref	1.02 (0.66–1.58)	**1.56 (1.00–2.44)**
Diabetes mellitus	Ref	0.57 (0.11–2.88)	1.87 (0.37–9.25)	Ref	2.85 (0.73–11.14)	**1.36 (1.64–11.23)**
Insulin resistance	Ref	0.12 (0.01–1.32)	0.49 (0.07–3.47)	Ref	0.91 (0.21–3.94)	3.10 (0.74–12.97)
Current smoker	Ref	0.92 (0.45–1.88)	0.82 (0.30–2.22)	Ref	2.65 (0.87–8.13)	1.75 (0.50–6.12)
Alcohol drinking	Ref	1.13 (0.76–1.70)	0.82 (0.46–1.45)	Ref	1.87 (0.94–3.75)	**2.13 (1.04–4.35)**
No control of salt intake	Ref	0.94 (0.87–1.03)	0.86 (0.73–1.02)	Ref	0.94 (0.87–1.02)	0.99 (0.92–1.08)

Values are PR (95% CI). Bold = p < 0.05. Abbreviations as in Online Table 1.

**Online Table 4:** Sensitivity analysis, rural Puno without data on family income (n = 191)

	Educational Attainment			Assets Index		
	None or Primary	Secondary	Higher	Lowest	Middle	Highest
Obesity	Ref	0.79 (0.17–3.81)	<0.01 (<0.01–<0.01)	Ref	0.29 (0.02–3.5)	1.0 (0.17–5.68)
Elevated WC	Ref	0.76 (0.50–1.16)	**3.13 (1.75–5.60)**	Ref	1.13 (0.77–1.65)	1.17 (0.77–1.77)
Hypertension	Ref	1.10 (0.37–3.30)	3.76 (0.77–18.44)	Ref	0.72 (0.32–1.62)	0.38 (0.08–1.75)
Low HDL-C	Ref	1.08 (0.76–1.53)	1.07 (0.25–4.57)	Ref	1.11 (0.79–1.57)	1.06 (0.71–1.58)
High TG	Ref	0.95 (0.43–2.11)	1.69 (0.41–7.03)	Ref	1.25 (0.54–2.84)	2.05 (0.82–5.10)
Diabetes mellitus	Ref	0.73 (0.06–8.57)	1.28 (0.23–7.19)	Ref	0.81 (0.20–3.35)	<0.01 (<0.01–<0.01)
Insulin resistance	Ref	<0.01 (<0.01–<0.01)	<0.01 (<0.01–<0.01)	Ref	0.44 (0.09–2.08)	0.20 (0.02–2.20)
Current smoker	Ref	0.88 (0.10–7.48)	9.63 (0.55–168.73)	Ref	2.36 (0.50–11.09)	0.66 (0.06–6.87)
Alcohol drinking	Ref	1.04 (0.49–2.21)	0.67 (0.18–2.45)	Ref	**3.76 (1.09–12.90)**	**4.28 (1.30–14.13)**
No control of salt intake	Ref	1.00 (0.97–1.04)	1.03 (0.98–1.08)	Ref	1.04 (1.00–1.08)	1.02 (0.97–1.07)

Values are PR (95% CI). Bold = p < 0.05. Abbreviations as in Online Table 1.
